# A system for inducing concurrent tactile and nociceptive sensations at the same site using electrocutaneous stimulation

**DOI:** 10.3758/s13428-012-0216-y

**Published:** 2012-07-18

**Authors:** Peter Steenbergen, Jan R. Buitenweg, Jörg Trojan, Esther M. van der Heide, Teun van den Heuvel, Herta Flor, Peter H. Veltink

**Affiliations:** 1 Biomedical Signals and Systems, Mira Institute for Biomedical Technology and Technical Medicine, University of Twente, Drienerlolaan 5, Postbus 217, Enschede, The Netherlands; 2 Department of Cognitive and Clinical Neuroscience, Central Institute of Mental Health, Medical Faculty, Heidelberg University Mannheim, Mannheim, Germany

**Keywords:** Electrocutaneous stimulation, Electric stimulation, Nociception, Touch, Quality of cutaneous perception, Reproducibility, Multi modal stimulation

## Abstract

Studies of the interaction between mechanoception and nociception would benefit from a method for stimulation of both modalities at the same location. For this purpose, we developed an electrical stimulation device. Using two different electrode geometries, discs and needles, the device is capable of inducing two distinct stimulus qualities, dull and sharp, at the same site on hairy skin. The perceived strength of the stimuli can be varied by applying stimulus pulse trains of different lengths. We assessed the perceived stimulus qualities and intensities of the two electrode geometries at two levels of physical stimulus intensity. In a first series of experiments, ten subjects participated in two experimental sessions. The subjects reported the perceived quality and intensity of four different stimulus classes on visual analogue scales (VASs). In a second series, we added a procedure in which subjects assigned descriptive labels to the stimuli. We assessed the reproducibility of the VAS scores by calculating intraclass correlation coefficients. The results showed that subjects perceived stimuli delivered through the disc electrodes as dull and those delivered through the needles as sharp. Increasing the pulse train length increased the perceived stimulus intensities without decreasing the difference in quality between the electrode types. The intraclass correlation coefficients for the VAS scores ranged from .75 to .95. The labels that were assigned for the two electrode geometries corresponded to the descriptors for nociception and touch reported by other researchers. We concluded that our device is capable of reliably inducing tactile and nociceptive sensations of controllable intensity at the same skin site.

**Electronic supplementary material:**

The online version of this article (doi:10.3758/s13428-012-0216-y) contains supplementary material, which is available to authorized users.

The skin contains receptors for various sensory modalities (Hollins, 2010; McGlone & Reilly, 2010). However, the integration of information from these modalities is at present poorly understood. In particular, the interaction between mechanoceptive and nociceptive information is an interesting topic from neurophysiological, clinical, and psychophysical perspectives. Mechanoception and nociception can originate at the same skin site and can be the result of the same physical stimulus, but these types of information are processed along separate neural pathways before being integrated into a single percept. A stimulation method that could allow control of tactile and nociceptive modalities at the same site would provide insights into the relation between these modalities—for instance, by allowing for study of detection thresholds and localization accuracy.

Tactile and nociceptive stimulation is commonly performed by using mechanical and laser stimulation. Using these methods to study the interaction between mechanoception and nociception can be challenging, especially if the stimulation site is to be varied during the experiment. When performing computer-controlled experiments with either of these methods, highly specialized equipment is required, such as a pneumatically driven mechanical stimulation array (Pott et al., 2010; Trojan et al., 2010) or a mirror–scanner system for laser stimulation (Trojan et al., 2006). Most importantly, mechanical stimulators would obstruct laser stimuli directed at the same stimulus site. We have developed a stimulation method that permits the stimulation of cutaneous tactile and fast nociceptive afferents at the same skin site (Steenbergen, Buitenweg, van der Heide, & Veltink, 2008). Our method employs electrocutaneous stimulation through a multichannel stimulator in combination with a compound electrode array. This approach allows for the application of complex spatiotemporal and multimodal stimulus patterns.

Because electrical stimulation directly activates afferent nerve fibers, rather than their sensory end structures (Bromm & Lorenz, 1998), such stimulation is often less selective in activating a specific afferent nerve fiber population than are other methods. The lack of selectivity in electrocutaneous stimulation can be overcome by choosing suitable electrode geometries. Electrical stimulation of A*β* afferents is easily achieved by using surface electrodes (see, e.g., Inui et al., 2003; Szeto & Saunders, 1982). Activation of A*δ* afferents in hairy skin can be achieved by using short needle electrodes that slightly penetrate the epidermis (Inui, Tran, Hoshiyama, & Kakigi, 2002; Inui, Tran, Qiu, Wang, Hoshiyama & Kakigi, 2002, 2003; Nilsson, Levinsson, & Schouenborg, 1997). This method is selective for A*δ* afferents when using limited stimulus currents, with the stimuli mostly being labeled as *pricking* or *tingling* (Mouraux, Iannetti, & Plaghki, 2010).

Most commonly, the perceived stimulus strength of electrical stimulation is varied by changing the stimulus current. Unfortunately, increasing the current through surface electrodes leads to a higher probability of undesired activation of A*δ* fibers, which is illustrated by the increase in the unpleasantness of stimuli with increasing amplitude (Janal, Clark, & Carroll, 1991). On the other hand, increasing the stimulus amplitude through needle electrodes leads to a higher probability of activating A*β* fibers (Mouraux et al., 2010). Instead, we chose to control the perceived stimulus strength by using pulse train modulation. Research by van der Heide, Buitenweg, Marani, and Rutten (2009) showed that it is possible to modulate perceived stimulus intensity by varying the number of applied stimulus pulses (NoP) in a pulse train. By repeatedly activating the same afferent nerve fibers, pulse train modulation mimics the way in which stimulus strength is coded in afferent nerve fibers following the regular activation of sensory end structures. By keeping the stimulus current constant, the same population of nerve fibers is activated at each stimulus level. Therefore, this method allows for varying the perceived stimulus strength while using a constant stimulus current that is at, or close to, the sensation threshold. This minimizes the probability of coactivation of cutaneous fiber populations other than the intended one.

For evaluating the stimulation method described above, a method was required that could detect small differences in perceived stimulus quality—for instance, due to a gradual increase in undesired fiber population activity when increasing the stimulus strength. The scientific literature provides only a small number of methods for assessing the perceived quality of cutaneous stimuli, none of which were suitable for our study. Janal et al. (1991) performed multidimensional scaling experiments on the dimensionality of painful and nonpainful electrocutaneous stimuli, and in a later study compared these stimuli to descriptive labels for painful and nonpainful stimuli (Janal, 1996). The results of this study cannot be used as the basis for an evaluation method of stimulus quality, since the authors influenced the painfulness of the stimuli by varying the stimulus voltages through the same electrode. Therefore, the dimensions of pain and intensity cannot be separated in their results. In Nahra and Plaghki (2003), subjects were asked to assign labels to painful laser stimuli. These stimuli, which included both A*δ* and C components, were reported mostly as *tingling* or *pricking.* These descriptors were greatly reduced after applying a block on all myelinated fibers; this block also increased the response latencies. This suggests that these labels are associated with perception of A*δ* fiber activity. Mouraux et al. (2010) used a similar labeling procedure for assessing the perception of laser stimuli as well as surface and needle electrode stimuli. *Pricking* and *tingling* were often assigned to both the needle electrode and laser stimuli. The surface electrode stimuli were often labeled as *touch* or *shock*. The labeling procedure used in both Nahra’s and Mouraux’s studies does not allow for detection of small shifts in quality that might be caused by varying the perceived stimulus strength. We therefore chose to use a VAS for perceived stimulus quality.

In the present study, we evaluated the perceptions elicited by the stimulation method described above. Our first aim was to determine whether our stimulus method was capable of successfully inducing qualities that could be associated with touch and nociception independently of each other. Secondly, we were interested whether pulse train modulation modifies the perceived stimulus strength without negatively affecting the quality of perception. Finally, we wanted to determine how reproducible these sensations are.

In a first series of experiments, we applied one- and five-pulse stimuli through both electrode types, leading to four different stimulus classes: needle electrodes with one and five pulses and disc electrodes with one and five pulses. Subjects reported the stimulus qualities and intensities on visual analogue scales (VASs). In a second series of experiments, a labeling procedure similar to the one used by Mouraux et al. (2010) was performed, in addition to the procedure employed in the first series. This labeling procedure allowed for comparing the results from the quality VAS with those from previous research. In each of the two series of experiments (first and second), subjects participated in two experimental sessions (A and B) on different days, which allowed for the assessment of reliability by calculating intraclass correlation coefficients (ICC).

## Method

### Procedure and material first series of experiments

The first series of experiments was performed in 2007 at Roessingh Research and Development (RRD) in Enschede, the Netherlands. The research protocol was approved by the Medical Ethical Board of RRD.

#### Subjects

For the first series of experiments, 13 subjects were recruited from the student population of the University of Twente. Three subjects were excluded because a bug in the experimental control software during one of the two sessions scrambled the order of the stimuli. The ten remaining subjects had a mean age of 26 years (standard deviation [*SD*] = 4, range 22 – 34 years), and two of the subjects were female. All subjects gave written informed consent prior to the first experimental session. For six of the subjects, the time between Experimental Sessions A and B was 7 days; for the other four, the intervals were 3, 14 (two subjects), and 21 days.

#### Apparatus

**Fig. 1:**
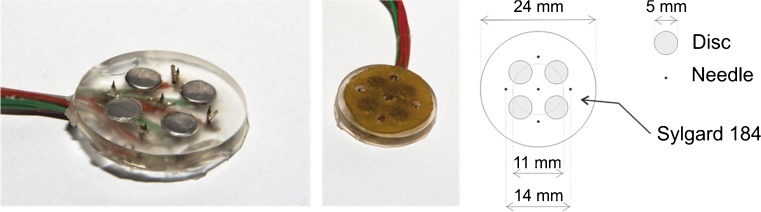
The compound electrode that was used for this study. (*Left*) Photograph without conducting pad. (*Middle*) Photograph with conducting pad. (*Right*) Schematic depiction. Each device consists of four disc and five needle electrodes mounted on a silicon base (Sylgard 184, Dow Corning)

The four flat discs of the compound electrode were punched out of a stainless-steel sheet; the five needle electrodes were made from stainless-steel sewing needles. The disc and needle electrodes are spread evenly over a disk with 2.4 - cm diameter (see Fig. 1, right panel). The needles protruded 0.5 mm from the electrode surface, and the discs were embedded in the surface. The base material for the compound electrode was Sylgard 184, a two-component silicone elastomer produced by the Dow Corning Corporation, Midland, Texas. The material can be created by mixing the supplied base and curing agents, which results in a viscous fluid. This was cast in a mold in order to shape the compound electrodes. A photograph and a schematic depiction of the compound electrode are presented in Fig. 1. To facilitate the electrical contacts between the disc electrodes and the skin, the compound electrode was covered with a conducting pad that covered the disc electrodes but had holes at the sites of the needle electrodes. The electrodes of the same type (needles and discs) were wired in parallel.

The stimulators used for generating the stimulus currents consisted of multiple channels that generated monophasic cathodic stimulus currents, the stimulus properties (amplitude, NoP, pulse width, and interpulse interval) of which could be configured for each channel independently.

The compound stimulation electrode was fixed with tape on the dorsal side of the left lower arm, halfway between the wrist and elbow. A counter electrode (anode) was fixed to the left wrist.

#### Stimuli

During the experiment, the perceived stimulus strength was modulated using pulse train modulation; one- and five-pulse stimuli were used. This resulted in four different stimulus classes: needle electrodes with one and five pulses, and disc electrodes with one and five pulses. These stimuli will be referred to, respectively, as N1, N5, D1, and D5. All of the pulses were 0.21-ms cathodic square waves. The interpulse interval (IPI) of the five-pulse stimuli was 5 ms, making the duration of these stimuli 21 ms. Stimulation with the needle and disc electrodes was performed at 130 % of the sensation thresholds (see below), with the thresholds determined for each separate session. For one subject, the amplitude of the needle electrode stimuli was increased to 160 % of threshold during the second session because the stimuli were not perceived at the default level. A sham stimulus was included as a fifth “stimulus” class in order to determine whether subjects responded to other cues than the stimulus when the stimulus was not perceived.

#### Determination of sensation thresholds

Separate sensation threshold currents of the disc and needle electrodes were determined at the start of each session using the method of limits (Gescheider, 1985). All of the stimuli were single, square-wave cathodic pulses with a pulse width of 0.21 ms. For the disc electrodes, the stimulus current was increased in steps of 0.3 mA, starting from zero. After subjects reported feeling a sensation, the current was lowered in steps of 0.1 mA until they stopped reporting a sensation. After this, the current was increased again in steps of 0.1 mA until the subjects reported feeling a sensation; this final detection current was recorded. For the needle electrodes, a similar method was used: The current was increased in steps of 0.10 mA, then lowered in steps of 0.05 mA, then increased in steps of 0.01 mA. The sensation threshold for disc electrodes is generally higher than that for needles, so the difference in step size for the two electrode types made sure that the method did not take very long for the disc electrodes or have a large overshoot for the needles. The procedure was repeated three times (trials) for each electrode type. The sensation threshold of each electrode type was calculated by averaging the recorded final stimulus current of the three trials.

#### VAS experimental procedure

**Fig. 2:**
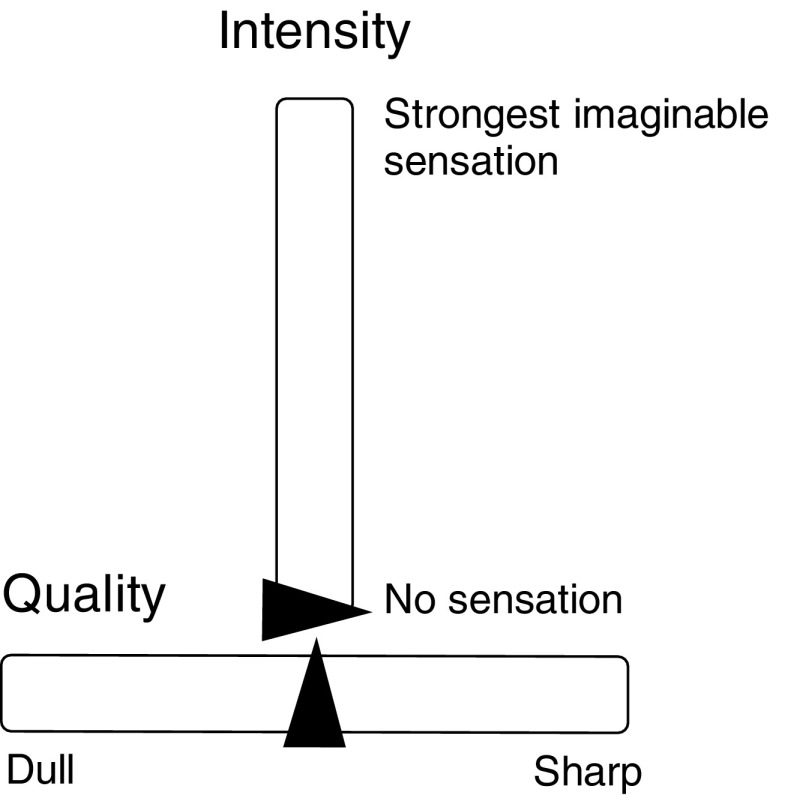
The VASs that were used for reporting the perceived quality and intensity following each stimulus. The black triangles represent the sliders that subjects manipulated to give their responses. For the first series of experiments, the texts were presented in Dutch. In the second series, they were presented in English. After reporting, the reports on the scales were converted to numbers ranging from 0 to 10. At the start of each trial, the sliders of the scales were preset at the bottom for intensity (*no sensation*, corresponding to an intensity score of 0) and in the middle for quality (neither *dull* nor *sharp*, corresponding to a quality score of 5)

During the experiments, the subjects were seated in front of a computer monitor. Following each stimulus, they were instructed to report the perceived stimulus intensity and quality by operating two VASs: one for the perceived quality of the stimuli, and one for the intensity (see Fig. 2). While the use of an intensity scale is similar to a VAS commonly used for the assessment of pain intensities, the quality scale had not been used before. The quality VAS was presented horizontally and ranged from *dull* to *sharp* (labeled in Dutch in this series but in English in the second series). We avoided labeling the extremes using terms that could be explicitly related to touch or nociception because we did not want to bias subjects toward reporting that the stimuli were tactile or nociceptive. Before use, the quality scale was preset in the middle because presetting the scale at one of the extremes might bias the reports toward either *dull* or *sharp*. The intensity VAS ranged from *no sensation* to *strongest sensation imaginable* and was preset at the bottom (*no sensation*). After each trial, the reports on the VAS scales were converted to numbers ranging from 0 to 10, corresponding to *no sensation* and *strongest sensation imaginable* for the intensity scale and *dull* and *sharp* for the quality scale. The subjects were not aware of the numeric values of their scores, since they were only presented with the scales and the anchors.

During the first series of experiments, the time between stimuli was fixed at 11 s. Subjects were told that they might not feel some of the stimuli and were instructed to do nothing following those. They were informed that leaving the scales at their preset value would be interpreted as the stimulus not having been perceived. In this case, the preset values were stored; during analysis, this combination of scores was used as an indicator of an undetected trial.

The five stimulus classes (N1, N5, D1, D5, and sham) were each applied 30 times. The stimulus sequence consisted of 30 different blocks in which the order of the five stimuli was randomized. The same sequence was used for each subject in both experimental sessions.

### Procedure and material second series of experiments

The second series of experiments was performed in 2011 at the Central Institute of Mental Health in Mannheim, Germany, and was approved by the Medical Ethics Committee II of the Medical Faculty Mannheim of Heidelberg University. The procedure for the second series of experiments was mostly the same as that for the first series, but aspects that differed between the second series and the first are described below.

#### Subjects

For the second series of experiments, 21 subjects were recruited from the staff and students of the Central Institute of Mental Health, two of which were excluded because they did not feel the disc electrode stimuli (for one subject, this was already the case during first threshold determination, and the other subject stopped feeling the disc stimuli shortly after the start of the VAS procedure). The remaining 19 subjects were on average 31 years old (*SD* = 6, range 21–52 years), and six were male. All subjects gave written informed consent prior to the first experimental session. The average time between Sessions A and B was 2 days (range 1–6 days).

#### Stimuli

Stimulation with the needle and disc electrodes was performed at 120 % of the sensation thresholds. The sham condition was omitted in the second series of experiments, since no subjects reported on the VAS following the sham stimuli in Series 1.

#### Determination of sensation thresholds

For Series 2, the sensation threshold determination was automated. The method for Series 1 had required the researchers to ask the subjects questions, which took a lot of time and created the possibility of biasing subjects because of the way in which questions were asked.

The sensation thresholds for the two electrode types were determined using a psychophysical threshold determination method consisting of multiple series of ascending stimuli. Subjects were instructed to press and hold a button; this initiated a trial consisting of a series of stimuli (one cathodic pulse with a pulse width of 0.21 ms) of ascending amplitude. The time between the stimuli was 1 s. The subjects were asked to release the button when they felt a sensation, which terminated the stimulus series. Following this, a logistic regression model was fitted to the series of detections (button releases) and misses (button not released). The sensation threshold was defined as the amplitude with a 50 % probability of detection. The threshold of each electrode was determined over ten trials. For the first trial at each of the two thresholds, the starting value was 0 mA; the increments were 0.1 mA for the needle electrodes and 0.5 mA for the disc electrodes. For the remaining trials, the starting value was half of the estimated threshold, and the increment was one eighth of this threshold. During each experimental session, the disc electrode threshold was determined first.

#### VAS experimental procedure

For the second series of experiments, the sham stimulus class was omitted from the VAS procedure. The procedure thus consisted of four stimulus classes (N1, N5, D1, and D5), each of which was applied in 30 randomized blocks. The randomization for this series was performed for each subject (the sequence was the same for both Sessions A and B in the same subject).

Each stimulus was preceded by a uniformly random waiting time of between 4 and 5 s. The subjects reported the sensations on the VASs after detecting each stimulus. After this, they clicked a “ready” button, which started the next stimulus cycle. When a subject failed to respond, the experimenter asked the subject to press the “ready” button without performing any reports. The new procedure allowed each subject as much time as needed to respond, without introducing an unnecessary waiting time.

#### Quality assessment procedure using labels

As a final part of the second series of experiments, each of the four stimuli that had been used during the VAS procedure was presented again. Each of the stimuli was repeated five times, after which the subject was asked to fill in a questionnaire based on the labels used by Mouraux et al. (2010) and Nahra and Plaghki (2003). Contrary to the VASs, which were presented with English labels, the questions were presented in German. For each stimulus class, subjects were asked whether they had detected any of the five stimulus presentations. If they had, they were asked to report the quality by assigning one or more of the following labels: *Leichte Berührung* (light touch), *Berührung* (touch), *elektrischer Schock* (shock), *prickelnd* (tingling), *stechend* (pricking), *warm* (warm), and *brennend* (burning).

### Data analysis

The data of the first and second experimental series were analyzed together, resulting in a data set containing 29 subjects. To correct for skewness, the sensation thresholds and stimulus currents were log transformed (using the natural logarithm). The effects of electrode type, (experimental) session, and series (of experiments) were analyzed by fitting linear mixed models (LMMs) using the Mixed procedure in SPSS 16.0. LMMs have a number of advantages as compared to a repeated measures ANOVA. The method allows for accounting for intersubject differences by including random effects; see West, Welch and Galecki (2007) for an introduction to this method. The factors Electrode Type and Session were modeled as repeated measures with a scaled identity covariance structure, and Series as a between-subjects factor. A random intercept for subjects was included in the model.

The VAS scores of undetected stimuli (5 for quality and 0 for intensity) were discarded. This included all sham stimuli in the first series of experiments, since none of those had been detected, and some of the other stimuli. The remaining scores were averaged by subject, electrode type, number of pulses (NoP), and session, resulting in four quality and four intensity scores for each session. The intensity scores were log transformed to correct for skewness. We assessed the effects of electrode type, NoP, session, and series by fitting an LMM using the Mixed procedure in SPSS 16.0. Electrode Type, NoP, and Session were modeled as repeated factors with a scaled identity covariance structure, and Series was modeled as a between-subjects factor. The model included a random intercept for subjects. Besides the four main effects, interaction effects were modeled for Series × Electrode Type, Series × NoP, Electrode Type × NoP, and Series × Electrode Type × NoP. Interaction effects were followed up by splitting the data over one of the interacting factors and fitting separate LMMs.

#### Reproducibility

Reproducibility of the sensation thresholds and of the quality and intensity VAS scores was assessed using intraclass correlation coefficients (ICCs) for each stimulus type. The appropriate ICC for the present study was ICC(1, *k*) (Shrout & Fleiss, 1979), which is calculated as follows:

$$ ICC\left( {i,k} \right) = \frac{{BMS - WMS}}{{BMS}} $$

Here, BMS is the between-subjects mean squares, and WMS is the within-subjects mean squares. An ICC of 1 (all variance is accounted for by differences between subjects) is interpreted as perfect reproducibility. If there are equal amounts of between- and within-subjects variability, the ICC(1, *k*) will be 0, which is interpreted as poor reproducibility. There is no objective limit above which an ICC represents good reproducibility; we will use .75 as a rule of thumb (Portney & Watkins, 2009).

ICCs of the session-averaged quality VAS scores as well as the session-averaged intensity VAS scores and thresholds were calculated, resulting in ten ICCs (two thresholds, four intensity scores, and four quality scores). Because the sensation threshold determination method for the first series of experiments was not based on a documented method, we assessed the reproducibility of this method by calculating an ICC using the three trials that were used. Each experimental session was considered as independent. This resulted in 20 sets of three repeated threshold determination trials for the disc and needle electrodes. Since each trial consisted of a staircase procedure in which subjects gave multiple responses, we can again use the ICC(1, *k*).

All calculations were performed in SPSS 16.0.

## Results

### Sensation thresholds

The sensation thresholds varied significantly between electrode types [*F*(1, 85) = *p* < 0.001], with the needle electrode sensation thresholds being 0.66 ± 0.37 (*M* ± *SD*) mA and the disc electrode thresholds being 2.82 ± 1.12 mA. The effects of session [*F*(1, 85) = 3.85, *p* = .053] and series [*F*(1, 27) = 4.09, *p* = .053] failed to reach significance by a small margin. The stimulus currents of the second series were significantly lower than those of the first series [*F*(1, 27) = 7.96, *p* = .009]. Just as for the sensation thresholds, the effect of electrode type was significant for stimulus currents [*F*(1, 85) = 431, *p* < .001], while the effect of session failed to reach significance [*F*(1, 85) = 3.64, *p* = .060].

The ICC of the threshold determination procedure of the first series of experiments, which was calculated over the three trials that were used for each threshold, was .91 (confidence interval .80–.96) for the needle electrodes and 1.00 (confidence interval .99–1.00) for the disc electrodes.

### Quality and intensity scores

**Fig. 3:**
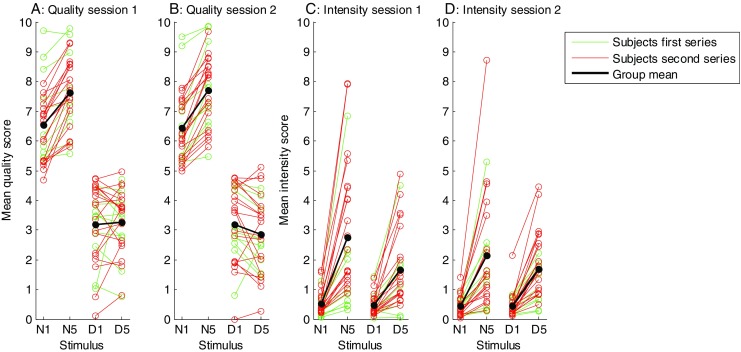
Averaged quality and intensity visual analogue scale (VAS) scores for each session and stimulus type per subject. The means for the whole population are indicated by bold lines. Subjects are color coded to distinguish between the first and second series of experiments. For the quality scores, the value 0 corresponds to the *dull* anchor on the quality VAS, and 10 corresponds to the *sharp* anchor. For the intensity scores, 0 corresponds to *no sensation* and 10 to *strongest sensation imaginable*. See Fig. 2 for a description of these scales

The quality and intensity scores for each subject, electrode type, NoP, and session are presented in Fig. 3, along with the mean scores of the whole subject population. Table 1 shows the results of the LMM analysis on these scores. Means and confidence intervals of the VAS scores of individual subjects are provided as supplementary materials.

**Table 1:** Linear mixed model analysis results on quality and intensity scores

	Quality			Intensity		
Factor	*df*	*F*	*p*	*df*	*F*	*p*
Electrode Type	1/196	573	<.001	1/196	5.57	.019
Number of Pulses (NoP)	1/196	8.16	.005	1/196	355	<.001
Session	1/196	0.441	.507	1/196	0.838	.361
Series	1/27	0.096	.759	1/27	2.06	.163
Series × Electrode Type	1/196	2.05	.154	1/196	0.562	.454
Series × NoP	1/196	0.716	.398	1/196	14.6	<.001
Electrode Type × NoP	1/196	13.1	<.001	1/196	4.68	.032
Series × Electrode Type × NoP	1/196	0.636	.426	1/196	0.903	.343

For the quality scores, there was a significant effect of electrode type, with the needle electrodes scoring more toward to the *sharp* end of the quality scale than did the disc electrodes. In addition, we found a significant NoP effect, as well as a significant Electrode Type × NoP interaction. We followed up on these effects by assessing the effect of NoP separately for each of the two electrode types using LMMs. All of the effects except electrode type were modeled, but we only tested the effect of NoP for each of the two electrode types. The effect of NoP on reported quality was significant for the needle electrodes [*F*(1, 81) = 114, *p* < .001] but not for the disc electrodes [*F*(1.81) = 1.08, *p* = .30]. For the needle electrodes, the reported quality was higher (sharper) for the N5 stimuli than for the N1 stimuli.

All subjects except one (for the disc electrodes of Session A) on average rated the five-pulse stimuli as being more intense than the one-pulse stimuli of the same electrode type. There was a significant effect of NoP on the reported intensity, with NoP = 5 stimuli being rated higher than the NoP = 1 stimuli. In addition, there was a significant effect of electrode type and an Electrode Type × NoP interaction effect. We followed up on this finding by analyzing the effect of electrode type for NoP = 1 and NoP = 5 separately using LMMs. The effect of electrode type on reported intensity was significant for the NoP = 5 stimuli [*F*(1, 81) = 8.73, *p* = .004], but not for NoP = 1 [*F*(1, 81) < 1.0, *p* = .87]. The N5 stimuli were rated with a higher intensity than were the D5 stimuli, but there was no difference in this respect between the N1 and D1 stimuli. Finally, we followed up the Series × NoP interaction effect on intensity scores by analyzing the effect of NoP for each series with separate LMMs. In both series of experiments, reported intensity was significantly influenced by NoP [*F*(1, 63) = 92.0, *p* < .001, for Series 1, and *F*(1, 126) = 340, *p* < .001, for Series 2], but the increase in intensity score between NoP = 1 and NoP = 5 was higher for Series 2 than for Series 1 (an increase of 1.01 for Series 1 and 1.89 for Series 2).

#### Labels

**Fig. 4:**
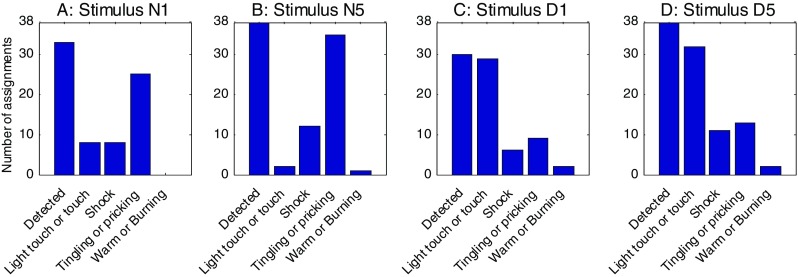
Results of the labeling procedure in the second series of experiments. Each graph (A–D) shows the results for one of the stimulus classes, aggregated over Sessions A and B, with the labels combined in categories. The “Detected” bar shows the number of sessions in which each of the stimulus classes was detected. The other bars show how many times each label category was applied to that stimulus class

The results of the labeling procedure of the second series of experiments are presented in Figs. 4A–4D. Nineteen subjects participated twice (Sessions A and B) in this part of the experiment, resulting in a total of 38 sessions in which the labeling procedure was performed. In all of these sessions, the N5 and D5 stimuli were detected. The N1 and D1 stimuli were missed in some cases, because the subjects sometimes had stopped feeling these stimuli in the course of the preceding VAS experiment. In order to determine in how many sessions labels that represented tactile or nociceptive sensations were assigned, we aggregated the label pairs *light touch*/*touch*, *tingling*/*pricking*, and *warm*/*burning*. We counted the number of times that either of the two labels of one category was reported. These aggregated scores showed that the majority of subjects reported the needle electrode stimuli as *tingling*/*pricking* and the disc electrode stimuli as *light touch*/*touch*. The number of assignments of these scores increased with increasing NoP. The *warm*/*burning* category was rarely reported. The *shock* label was reported for all stimulus classes in a small number of sessions.

#### Reproducibility

The ICCs for the thresholds and the quality and intensity scores are listed in Table 2. All ICCs except for the disc electrode sensation threshold were .75 or higher. The VAS scores for each electrode type had higher ICCs than the respective sensation thresholds. Although most of the ICCs had lower confidence boundaries beneath .75, the consistently high ICC estimates suggest good reproducibility overall. Separate ICCs for both series of experiments are provided in the supplementary materials.

**Table 2:** Intraclass correlation coefficients with 95 % confidence intervals of the pooled thresholds and visual analogue scale ratings in both series of experiments

	Needle Electrodes		Disc Electrodes	
	NoP = 1	NoP = 5	NoP = 1	NoP = 5
Threshold^1^	.77 [.50, .89]		.59 [.13, .81]	
Quality	.88 [.75, .94]	.93 [.85, .97]	.95 [.90, .98]	.85 [.68, .93]
Intensity^1^	.75 [.48. .88]	.84 [.65, .92]	.86 [.71, .94]	.80 [.58, .91]

NoP, number of pulses. ^1^Thresholds and mean intensity scores were log transformed.

## Discussion

We collected quality and intensity VAS scores for stimuli applied with our compound electrode array. Needle and disc electrode stimuli with two intensity levels were delivered. Subjects participated in two experimental sessions, which enabled analysis of the reproducibility of the outcome measures. The reports on the quality VAS showed that stimuli applied through the needle and disc electrodes elicited clearly distinguishable dull and sharp sensations. A larger number of pulses in the stimuli was demonstrated to increase the reported intensity of the stimuli without any detrimental effects on the quality scores. The ICCs of the quality and intensity scores indicated good reproducibility of the perceived stimulus qualities and intensities induced by our stimulation method.

Our subjects reported the perceived stimulus quality on a continuous scale ranging from *dull* to *sharp*. They generally reported disc electrode stimuli to be on the dull half of the scale and needle electrode stimuli to be on the sharp half. These scores by themselves do not provide evidence that the disc electrode stimuli led to tactile and the needle-induced stimuli to nociceptive sensations. However, the assignments of the qualitative labels (predominantly *tingling* and *pricking* to the needle electrode stimuli and *light touch* and *touch* to the disc electrode stimuli) strongly suggest that subjects associated sensations at the *dull* anchor point in our quality VAS with a tactile quality and the *sharp* anchor with nociception.

The present study is the first in which the effect of pulse train modulation (PT) is explored for preferential electrical stimulation of nociceptive and tactile afferents. Although van der Heide et al. (2009) studied PT in detail, the stimulation electrode that they used recruited a mixed population of afferents. Our results suggest that PT is capable of modulating the perceived intensity of both nociceptive and tactile stimuli. We did not study the effect of NoP over the range that van der Heide et al. had used, and therefore we do not know whether the saturation in intensity for NoP > 7 that they found exists for both nerve fiber populations. In the present study, PT influenced the intensity scores of the needle electrode stimuli more strongly than those of the disc electrode stimuli. This may be attributed to differences in which action potential frequencies code for stimulus strength in tactile and fast nociceptive afferents.

Since the dull and sharp qualities were presented on the same VAS, subjects were not given the opportunity to report a quality containing both a dull and a sharp component. The small number of nociceptive labels assigned to the disc electrode stimuli—and, vice versa, of tactile labels assigned to the needles—suggests that this situation was rare in our study. Recording dullness and sharpness of stimuli using a VAS without the attributes being mutually exclusive would be possible if separate scales were used for dullness and sharpness. This procedure could be extended to include any number of qualitative attributes. This would combine the advantage of using a continuous quality scale, which records small shifts in perceived quality, with the advantage of a labeling procedure, which gives the possibility of recording multiple qualitative aspects of the same stimulus. Before designing a method like this, it would be useful to gather more knowledge on the parameter space of the quality of cutaneous perception, for instance by using a multidimensional scaling procedure.

Because we wanted to determine the reproducibility of the reported qualities and sensations of the stimuli, each subject participated in two experimental sessions, and ICCs were calculated. The VAS-score ICCs demonstrate that the stimuli through the electrode array led to highly reproducible sensations. The ICCs of the VAS scores were all higher than the ICCs of the sensation thresholds. This suggests that the reproducibility of the quality and intensity of the sensations appears to be quite robust to small changes in the stimulus currents. Although the stimulus currents used in the two series of experiments were significantly different, nine out of the ten ICCs for the pooled data lie between the ICCs calculated separately for both series of experiments (see the supplementary materials). This indicates that pooling the data did not lead to inflated ICCs through increased intersubject variability caused by differences in the experimental procedures.

Although only single phasic stimuli were generated in this study, the multichannel stimulators that were used would allow for the generation of more complicated stimulus patterns. Stimulators with any number of channels can be built and used with multiple compound electrode arrays. This system can be used for a range of experimental paradigms. First of all, the tactile and nociceptive content in a stimulus can be varied in a controlled manner by applying pulse trains containing a mixture of needle and disc electrode pulses, the proportion of which can be varied. Secondly, when multiple electrode arrays are used, a comparison of the spatial perception of touch and nociception can be made in a single experiment. Mancini, Longo, Iannetti, and Haggard (2010) performed a within-subjects comparison of the reported locations of touch and fast and slow nociception. Because of the stimulus methods employed (mechanical and laser), each modality had to be applied in a separate experiment. The use of compound electrode arrays in combination with multichannel stimulators allows for comparisons within a single experiment in which the stimuli of the two modalities can be randomized. A third application would be the study of spatiotemporal, multimodal stimulus patterns. Any real-life stimulus involves multiple modalities over a length of time, but it is poorly understood how these aspects are integrated into a single percept. Studying spatiotemporal sensory phenomena may provide important insights on this topic, for instance through studying the saltation effect (Trojan et al., 2006).

Although our results show that the stimuli delivered through our compound electrode array correspond well to tactile and nociceptive sensations, we do not have proof that the two electrode geometries activate tactile and nociceptive afferents selectively. Our needle electrodes were similar to the one used by Mouraux et al. (2010), which was demonstrated to be selective for stimulus currents comparable to ours in magnitude. For the disc electrodes, we do not have this kind of information, and we therefore have to take into account the possibility that they may activate some nociceptive afferents besides the intended tactile afferents.

Our compound electrode array offers the possibility of studying touch and nociception arising from the same site. However, this is only a small fraction of the cutaneous sensory modalities in existence. Some of these modalities are not stimulated by our method at all; this includes all modalities whose information is transmitted through C-fiber afferents, which are activated by stimulus currents higher than those of the myelinated cutaneous afferents (Malmivuo & Plonsey, 1995). Furthermore, our activation of tactile fibers does not discriminate between afferents connected to different types of receptors.

We conclude that the use of disc surface electrodes and needle electrodes in combination with our multichannel stimulators is capable of eliciting two distinguishable sensations at the same skin site. These sensations correspond to tactile and nociceptive modalities, and the perceived quality of them is reproducible. The perceived strength of the stimuli can be varied without detrimental effects on the perceived qualities. Ours is therefore a promising method for studying the interaction between touch and nociception arising from the same skin site, for instance by studying spatial perception of cutaneous stimuli and sensation thresholds. This may give rise to new insights about the ways in which the various cutaneous sensory modalities interact.

## Electronic Supplementary Materials

Below is the link to the electronic supplementary material.

ESM 1 (DOC 84 kb)

## References

[CR1] Bromm B, Lorenz J (1998). Neurophysiological evaluation of pain. Electroencephalography and Clinical Neurophysiology.

[CR2] Gescheider GA (1985). Psychophysics: Method, theory, and application.

[CR3] Hollins M (2010). Somesthetic senses. Annual Review of Psychology.

[CR4] Inui K, Tran TD, Hoshiyama M, Kakigi R (2002). Preferential stimulation of Adelta fibers by intra-epidermal needle electrode in humans. Pain.

[CR5] Inui K, Tran TD, Qiu Y, Wang X, Hoshiyama M, Kakigi R (2002). Pain-related magnetic fields evoked by intra-epidermal electrical stimulation in humans. Clinical Neurophysiology.

[CR6] Inui K, Tran TD, Qiu Y, Wang X, Hoshiyama M, Kakigi R (2003). A comparative magnetoencephalographic study of cortical activations evoked by noxious and innocuous somatosensory stimulations. Neuroscience.

[CR7] Janal MN (1996). Concerning the homology of painful experiences and pain descriptors: A multidimensional scaling analysis. Pain.

[CR8] Janal MN, Clark WC, Carroll JD (1991). Multidimensional scaling of painful and innocuous electrocutaneous stimuli: Reliability and individual differences. Perception & Psychophysics.

[CR9] Malmivuo J, Plonsey R (1995). Principles and applications of bioelectric and biomagnetic fields.

[CR10] Mancini F, Longo MR, Iannetti GD, Haggard P (2010). A supramodal representation of the body surface. Neuropsychologia.

[CR11] McGlone F, Reilly D (2010). The cutaneous sensory system. Neuroscience and Biobehavioral Reviews.

[CR12] Mouraux A, Iannetti GD, Plaghki L (2010). Low intensity intra-epidermal electrical stimulation can activate Adelta-nociceptors selectively. Pain.

[CR13] Nahra H, Plaghki L (2003). The effects of A-fiber pressure block on perception and neurophysiological correlates of brief non-painful and painful CO2 laser stimuli in humans. European Journal of Pain.

[CR14] Nilsson HJ, Levinsson A, Schouenborg J (1997). Cutaneous field stimulation (CFS): A new powerful method to combat itch. Pain.

[CR15] Portney LG, Watkins MP (2009). Foundations of clinical research: Applications to practice.

[CR16] Pott PP, Kamping S, Bomba IC, Diesch E, Flor H, Schwarz MLR (2010). An MR-compatible device for automated and safe application of laser stimuli in experiments employing nociceptive stimulation. Journal of Neuroscience Methods.

[CR17] Shrout PE, Fleiss JL (1979). Intraclass correlations: Uses in assessing rater reliability. Psychological Bulletin.

[CR18] Steenbergen P, Buitenweg JR, van der Heide EM, Veltink PH, van der Sloten J, Verdonck P, Nyssen M, Haueisen J (2008). Characterisation of a bimodal electrocutaneous stimulation device. 4th European Conference of the International Federation for Medical and Biological Engineering (IFMBE Proceedings.

[CR19] Szeto AYJ, Saunders FA (1982). Electrocutaneous stimulation for sensory communication in rehabilitation engineering. IEEE Transactions on Biomedical Engineering.

[CR20] Trojan J, Stolle AM, Kleinböhl D, Mørch CD, Arendt-Nielsen L, Hölzl R (2006). The saltation illusion demonstrates integrative processing of spatiotemporal information in thermoceptive and nociceptive networks. Experimental Brain Research.

[CR21] Trojan J, Stolle AM, Mršić Carl A, Kleinböhl D, Tan HZ, Hölzl R (2010). Spatiotemporal integration in somatosensory perception: Effects of sensory saltation on pointing at perceived positions on the body surface. Frontiers in Perception Science.

[CR22] van der Heide EM, Buitenweg JR, Marani E, Rutten WL (2009). Single pulse and pulse train modulation of cutaneous electrical stimulation: A comparison of methods. Journal of Clinical Neurophysiology.

[CR23] West BT, Welch KB, Galecki AT (2007). Linear mixed models: A practical guide using statistical software.

