# Supplementary material for: A system for inducing concurrent tactile and nociceptive sensations at the same site using electrocutaneous stimulation
Source: Behav Res Methods. 2012 Jul 18;44(4):924–33. doi: 10.3758/s13428-012-0216-y (PMC3509324; doi:10.3758/s13428-012-0216-y)
Supplement: Supplementary file 1 — (DOC 84 kb) [file 13428_2012_216_MOESM1_ESM.doc]

**Supplementary material**

*A system for inducing concurrent tactile and nociceptive sensations at the same site using electrocutaneous stimulation*

*Peter Steenbergen, Jan R. Buitenweg, Jörg Trojan, Esther M. van der Heide, Teun van den Heuvel, Herta Flor & Peter H. Veltink*

**Contents**

Page 2-4: Mean and standard deviations of the quality and intensity VAS scores of individual subjects

Page 5: Separate Intraclass Correlation Coefficients calculated for the first and second series of experiments

**Intraclass correlations calculated separately for experiment series 1 and 2**

| *ICCs with 95% CI of the thresholds and VAS of experiment series 1* | | | | |
| --- | --- | --- | --- | --- |
|  | **Needle electrodes** | | **Disc electrodes** | |
|  | NoP=1 | NoP=5 | NoP=1 | NoP=5 |
| Threshold | 0.66 [-.27, .91] |  | 0.82 [.32, .95] |  |
| Quality | 0.94 [.78, .99] | 0.96 [.83, .99] | 0.91 [.66, .98] | 0.78 [.18, .95] |
| Intensity | 0.71 [-.11, .93] | 0.96 [.85, .99] | 0.69 [-.19, .92] | 0.62 [-.42, .91] |

| *ICCs with 95% CI of the thresholds and VAS of experiment series 2* | | | | |
| --- | --- | --- | --- | --- |
|  | **Needle electrodes** | | **Disc electrodes** | |
|  | NoP=1 | NoP=5 | NoP=1 | NoP=5 |
| Threshold | 0.76 [.36, .91] |  | 0.29 [-.85, .73] |  |
| Quality | 0.76 [.37, .91] | 0.89 [.71, .96] | 0.97 [.92,.99] | 0.89 [.68, .95] |
| Intensity | 0.67 [.15, .88] | 0.74 [.32, .90] | 0.92 [.80, .97] | 0.89 [.70, .96] |
